# A Systematic Review and Single-center Experience of Azygos Vein Defibrillator Lead Insertion

**DOI:** 10.19102/icrm.2022.130502

**Published:** 2022-05-15

**Authors:** Ying Chi Yang, Suchith Shetty, Ketan Koranne, Aaqib Malik, Michael Giudici

**Affiliations:** ^1^Department of Internal Medicine, University of Iowa Health Care, Carver College of Medicine, Iowa City, IA, USA; ^2^Department of Cardiology, MercyOne North Iowa Medical Center, Mason City, IA, USA; ^3^Department of Cardiology, Westchester Medical Center and New York Medical College, Valhalla, NY, USA

**Keywords:** Azygos vein defibrillator lead, high defibrillatory threshold, implantable cardioverter-defibrillator

## Abstract

Defibrillation threshold (DFT) testing is performed in individuals with higher predicted risks of defibrillation failure. Many strategies have been explored to overcome the challenge of high DFT, including an insertion of a defibrillator lead into the azygos vein. We performed a systematic review of the literature to evaluate the safety and efficacy of azygos vein implantable cardioverter-defibrillator insertion for high DFT combined with the analysis of a single-center experience of the procedure at our institution. The literature search was performed in PubMed and Embase from database inception to December 2020 to identify all case reports and case series related to azygos vein defibrillator lead insertion. Our search identified 291 records. After excluding duplicate studies and those without DFT thresholds and non-azygos vascular destinations, 12 studies (23 cases) were reviewed from the current database. We also conducted a retrospective analysis of 5 cases performed at our institution, and a total of 28 patients were included in our final analysis. The mean age of the pooled cohort was 47 years (range, 17–88 years). Men composed 92% of the total cases, and the average body mass index was 34 kg/m^2^. The mean ejection fraction (EF) was 25%, with 78% having non-ischemic cardiomyopathy. The left axillary (36%) or subclavian (48%) vein was the common percutaneous access point. The mean duration of azygos vein access and lead delivery was 22 min (range, 13–60 min). The average DFT prior to azygos coil insertion was 35 J (range, 20–45 J). Fifty-seven percent of cases achieved substantial DFT improvement, whereas 18% achieved relative improvement compared to the pre-procedural threshold. No immediate or remote procedure-related complications were reported in 24 cases where data were available. During an average follow-up period of 18 months, 5 patients had ventricular arrhythmic events requiring device therapy and 4 had successful cardioversion from the device. One patient died from cardiac arrest with variable device therapies of both unsuccessful and successful events. In conclusion, azygos vein defibrillator lead insertion has a considerable rate of success, ease of vascular access with minimal procedural time, and lower risks and complications. Larger studies and longer follow-up periods are warranted to establish its efficacy and safety.

## Introduction

Defibrillation threshold (DFT) is defined as the minimal amount of energy required to successfully terminate the induced ventricular fibrillation (VF) by the implantable cardioverter-defibrillator (ICD) on 2 consecutive occasions.^1^ Many experts recommend testing DFT in a select group of patients with high predicted risks of defibrillation failure. High DFT is defined when there is no minimal safety margin of 10 J below the generator maximal output. Many strategies have been explored to overcome the challenge, including the insertion of a defibrillator lead into the azygos vein. The current literature for the efficacy and safety of azygos defibrillator lead insertion in the setting of high DFT is only limited to case reports and case series. In this study, we performed a systematic review of the literature to analyze the cumulative evidence of its efficacy and safety profile along with the demographics of the patients requiring such a procedure. We also included a retrospective analysis of outcomes of all 5 cases performed at our institution to the present date.

## Methods

**Figure 1:**
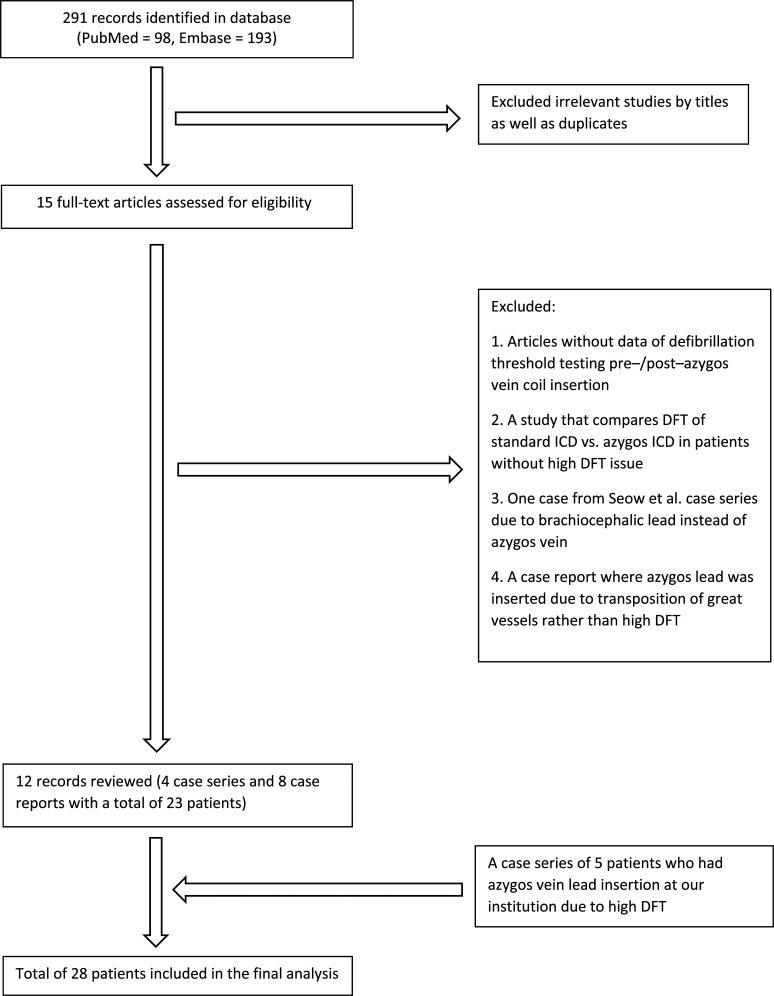
Study selection process according to Preferred Reporting Items for Systematic Reviews and Meta-analyses. *Abbreviations:* DFT, defibrillation threshold; ICD, implantable cardioverter-defibrillator.

A comprehensive review of the literature was performed, searching PubMed and Embase from database inception to December 2020 to identify all studies related to azygos defibrillator lead insertion for high DFT. We utilized the following search terms “azygos lead” or “azygos coil” or “azygous lead” or “azygous coil.” Two investigators (Y. Y. and S. S.) independently searched for potentially relevant articles. A total of 291 records were initially identified **(Figure 1)**. After excluding the duplicates and irrelevant studies by their title, we assessed 15 full-text articles for their eligibility. We included articles with sufficient data of DFT values prior to and after azygos coil insertion. We excluded a study that directly compared DFT between standard and azygos defibrillator leads in patients without known high DFT issues. We excluded 1 patient from the Seow et al.^2^ case series as their lead was inserted into the brachiocephalic vein. We also excluded 1 case report where the azygos lead was inserted due to the transposition of the great vessels rather than high DFT. The Preferred Reporting Items for Systematic Reviews and Meta-analyses guideline was followed for the entire selection process. In addition, we performed a thorough chart review of our electronic medical records and included all 5 patients who underwent azygos vein defibrillator lead insertion at our institution in the setting of high DFT. We then performed a descriptive analysis after pooling the individual cases to form a study cohort.

## Results

Thirteen studies with a total of 28 patients, including our center’s cases, were analyzed in this pooled cohort. The largest case series included 7 patients (Cooper et al.^3^, followed by the one at our center with 5 patients. The mean age of the pooled cohort was 47 years (range, 17–88 years). Men composed 92% of the study group. Body mass index (BMI) data were available in the 2 largest case series, with an average of 34 kg/m^2^ (range, 21–56 kg/m^2^). Left ventricular (LV) ejection fraction (LVEF) was reported in 22 cases, and its median was 25% (range, 15%–40%) with 78% of patients having non-ischemic cardiomyopathy (NICM). LV end-diastolic diameter (LVEDD) was reported in 13 cases, with an average of 7.3 cm (range, 4.5-9.0 cm). Among 18 cases that included anti-arrhythmic data, 28% were on amiodarone therapy. Left axillary and subclavian veins were the most favored percutaneous access sites in 36% and 48%, respectively. Duration from vascular access to azygos lead delivery was reported in 13 cases, and the mean duration was 22 min (range, 13–60 min). Prior to azygos lead insertion, 96% in the study had failed initial DFT testing owing to either unsafe DFT or unsuccessful cardioversion with the maximal generator’s capacity. The mean value of maximal DFT tested prior to azygos lead insertion was 35 J (range, 20–45 J). Fifty-seven percent of cases achieved substantial DFT improvement (decreased by ≥10 J following the procedure), whereas 18% achieved an improvement of less than 10 J in DFT compared to the pre-procedural threshold. Eighteen percent of cases who previously failed to be cardioverted with the maximal generator’s capacity had successful cardioversion from the azygos lead defibrillator despite the lack to keep a safe DFT margin **(Tables 1 and 2)**. One case failed show improved DFT regardless of the procedure. One case from the series of Cesario et al.^4^ series received an azygos lead not because of high DFT but due to a prior left pectoral ICD pocket infection and a right shoulder gunshot wound. The DFT in that case was found to be as low as 10 J with the azygos coiling. No immediate or remote procedure-related complications were reported in 24 cases (86%) where data were available. One patient experienced the lead migration to the proximal end of the azygos vein shortly after the procedure, but it was repositioned and successfully secured by a vascular plug. During an average follow-up period of 18 months (range, 4–60 months), 5 patients had ventricular arrhythmic events requiring device therapy and 4 of them had successful cardioversion from the device. One patient died from cardiac arrest after receiving both successful and unsuccessful device therapies.^5^

### Implant technique

The generator was temporarily removed with an incision to the pocket. The left axillary or left subclavian vein was accessed via the pocket using the radiographic landmarks. A J guidewire was introduced to the central circulation, and a long peel-away sheath was then advanced to the left subclavian vein over the guidewire. The azygos vein was cannulated with a 90° subselector catheter, and the Wholey wire (Medtronic, Minneapolis, MN, USA) was advanced to the distal portion of the vein. The subselector catheter was then removed, and the peel-away sheath was advanced to the mid-portion of the azygos vein over the guidewire. The defibrillator lead was then advanced through the peel-away sheath and secured in the mid-portion of the azygos vein. Finally, the peel-away sheath was removed, and the lead was secured in the pocket with standard sutures.

## Discussion

**Figure 2:**
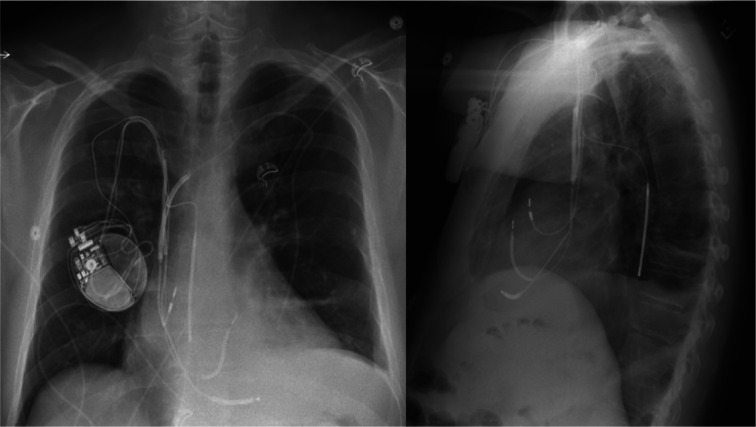
Chest X-rays showing an azygos defibrillator lead located posterior to the left ventricle.

DFT testing has historically been an essential component of ICD implantation. However, in recent years, it has fallen out of being a universal protocol during implantation of left-sided transvenous devices with the emergence of data, which have shown no mortality benefits of the test. Nonetheless, many experts still recommend testing DFT in a select group of patients who carry higher predicted risks of elevated DFT as the benefit/risk ratio of testing may be favored in those populations. The Ejection Fraction Secondary Prevention as the ICD Indication, Age, Gender, and Amiodarone (EF-SAGA) risk scoring model is among the tools used when determining who may benefit from DFT testing. It stratifies risks of high DFT based on the male gender, age < 60 years, EF < 20%, secondary prevention as the indication for ICD, and amiodarone therapy. Despite the variable consensus, patients with a prior history of failed device therapy, right-sided implants, and hypertrophic cardiomyopathy (HCM) are recommended for routine DFT testing. In general, high DFT is found in 6%–12% of ICD candidates.^6,7^ DFT is the minimal amount of energy delivered by the device to successfully terminate induced VF on 2 consecutive occasions.^1^ High DFT is defined when there is no minimal safety margin of 10 J below the generator maximal output. Many strategies have been explored to overcome the challenge of high DFT. Implanting subcutaneous arrays is among the alternatives to improve DFT by redirecting the shock vectors.^8^ However, its utility is limited by higher infection risks, patient’s discomfort, and lead fractures. Moreover, subcutaneous arrays are not compatible with right-sided generators due to the misalignment of vectors. Repositioning of the right ventricular (RV) lead and alternation of shock waveforms are also considerable alternative strategies.^9^ Different techniques, such as inserting an additional defibrillator lead to the superior vena cava, coronary sinus, or hemi-azygos or azygos vein, have been sporadically described in the literature. Over the last few decades, azygos lead defibrillation lead insertion has brought much more attention to the electrophysiologist community compared to other vascular destinations. The technique allows the discharge coil to locate immediately posterior to the heart, creating a more favorable vector across the LV myocardium to the can **(Figure 2)**.^3,4^ However, the impedance of azygos coils is often higher than that of conventional RV leads, and so we hypothesize that the mechanism behind its improvement in DFT is from the optimal alignment of defibrillation vectors rather than an alternation of tissue resistance. Cooper et al.^3^ conducted a study to evaluate DFT between conventional and azygos lead ICDs in the patient population without high DFT and found no benefits between the 2 groups. However, azygos lead insertion did offer DFT improvement in patients with elevated DFT in a case series published by the same author. Based on the accumulated data from the published case reports and case series of the technique, azygos vein defibrillator lead insertion has shown substantial improvement in patients with high DFT. We also analyzed the clinical characteristics and outcomes of patients from the study group compared to the conventional ICD candidates. In the most recent meta-analysis of 6 randomized controlled trials by Khan et al.,^10^ who studied the efficacy of ICDs in NICM, the medium age of conventional ICD recipients was 60 years and men composed 73% of the cohort. In our pooled cohort, the mean age was 47 years, with 92% being men. We also found that our study group has higher LVEDD parameters although we do not have data about LV mass. The patient population requiring azygos leads was found to be younger, to be substantially male, and to have increased LVEDD. In general, men have a higher risk of receiving any type of ICD, but data from our study cohort unveiled an even stronger correlation of men to receive an azygos lead ICD. Male gender is known to be associated with greater LV mass and increased LVEDD. In addition, those parameters also peak between the ages of 20–50 years.^11^ Based on our analysis, a substantial proportion of male gender, younger age, and increased LVEDD in the study group would support the hypothetical correlation between those parameters and high DFT leading to azygos lead insertion. The average BMI of the population who received the procedure was 34 kg/m^2^, which is also significantly higher than the mean BMI of 27 kg/m^2^ for conventional ICD recipients.^10,12^ Obesity has been accepted as an independent risk factor of high DFT.^3,13^ We hypothesize that higher BMI is associated with a higher risk of elevated DFT due to the increase in distance between the defibrillator can and RV defibrillator lead as well as the increase in tissue resistance from adipose tissues. We also noted that the majority of our candidates have NICM, and the etiologic diversity of NICM (dilated, hypertrophic, or restrictive cardiomyopathy) may lead to an increased risk of high DFT from various factors, such as electrode distance, tissue resistance, and impedance. In our study, 2 cases were related to HCM. HCM is often associated with high DFT, which is presumed from the anterior and superior displacement of the RV due to the hypertrophic LV mass compromising the defibrillation vector from the RV apex to the can across the LV myocardium.^5^ LVEF and amiodarone therapy are among the DFT-influencing factors, which may theoretically pose greater chances of azygos vein coiling.^14^ However, the mean LVEF of our study group was found to be 25%, which is comparable to that of the population who received conventional ICDs (23%).^10^ In general, 15% of ICD recipients are on amiodarone therapy.^15^ We found the higher percentage of amiodarone use to be 28% in our study population. Overall, the baseline characteristics of our study population who received azygos lead insertion were comparable to the group of high DFT risks described in the current literature. In terms of technicality, azygos defibrillator lead insertion is not much more complex than the conventional ICD implantation process. It utilizes the same subclavian access as used in the conventional ICD procedures in a majority of cases. The average duration of lead delivery into the azygos vein in the study cohort was 23 min, supporting the technical feasibility of azygos defibrillator lead addition.^16^ The most recent study of ICD efficacy in NICM showed that the cardiovascular mortality rate in the NICM population was 13.8% over an average follow-up of 68 months.^12^ The cardiac mortality rate of our cohort was found to be 3.8%, which is lower compared to that of patients with conventional ICDs. However, the follow-up duration of our study was significantly shorter. Nonetheless, the cardiovascular mortality rate of azygos vein lead insertion is comparable to that of the conventional procedure, even with the adjustment of follow-up duration.

Our study has several limitations. First, this study is a systematic review of published case reports and case series without the inclusion of larger clinical trials, thus limiting our study cohort to a smaller sample size. Second, not all studies included the baseline characteristics. Third, follow-up periods varied substantially between the included studies. Fourth, only 28% of the study population experienced clinical arrhythmia to trigger device therapy, limiting the data of one of the most important outcomes in this study. Lastly, due to the non-randomized nature of the available data, we were unable to make a temporal association or draw firm conclusions on the outcomes of azygos vein defibrillator insertion and post-implant successful device therapy.

## Conclusion

Data from our small cohort show that azygos vein defibrillator lead insertion has a considerable rate of success, ease of vascular access with minimal procedural time, and lower risks and complications. Nevertheless, larger studies and longer follow-ups are warranted to establish its efficacy and safety.

## Figures and Tables

**Table 1:** Clinical Characteristics and Outcomes of the Pooled Cohort for Azygos Vein Lead Implantable Cardioverter-Defibrillator

Study/Author	Our Institution	Seow et al.^2^	Cooper et al.^3^	Cesario et al.^4^	Bar-Cohen et al.^5^	Kommuri et al.^17^	Moran et al.^18^	Yamada et al.^19^	Jastrzębski et al.^20^	Camanho et al.^21^	Rahaby et al.^22^	Dayer et al.^23^	Pugh et al.^24^
No. of cases	5	2	7	3	1	3	1	1	1	1	1	1	1
Age (years)	45 (29–62)	73 (69–77)	37 (18–56)	53 (44–58)	17	53 (30–88)	NR	72	34	37	44	56	61
Gender	M	1M, 1F	M	M	F	M	M	M	M	M	M	M	M
BMI (kg/m^2^)	37 (30–53)	NR	32 (21–56)	NR	NR	NR	NR	NR	NR	NR	NR	NR	NR
Mean LVEF%	28 (15–35)	38 (35–40)	23 (17–27)	25 (20–30)	NR	25 (20–30)	NR	NR	NR	17	20	NR	NR
Etiology of heart failure	4/5 NICM	ICM	6/7 NICM	2/3 NICM	NICM	NICM	ICM	NICM	NICM	NICM	NICM	NICM	NICM
Amiodarone therapy	2/5	NR	2/7	0/3	No	NR	No	NR	NR	NR	NR	NR	Y
Left ventricular end-diastolic diameter (cm)	7.1 (6.4–7.7)	NR	7.2 (4.5–8.4)	NR	NR	NR	NR	NR	NR	9	NR	NR	NR
Percutaneous access	4 LAX, 1 LSC	LAX	7 SC	2 LAX, 1 LF	NR	LAX	RSC	LSC	NR	LAX	LSC	NR	LSC
Duration from vascular access to azygos lead delivery (min)	NR	15–20	15 (13–18)	30 (20–40)	NR	NR	NR	NR	NR	NR	NR	< 60	NR
Procedural complication	None	None	None	None	Lead Migration	None	None	None	NR	NR	NR	None	None
Maximal DFT tested with prior ICD system	39 J (30–45 J)	20 J, 20 J*	−10 J below the maximal output	36 J (41–31 J)	36 J	34 J (31–41 J)	35 J	36 J	40 J	35 J	25 J	35 J	41 J fail
DFT after azygos lead insertion	31 J (21–41 J)	10 J, 10 J	4 safe margin > 10 J, 2 safe margin < 9 J, 1 no safe margin	23 J (10–31 J)	29 J	24 J (21–31 J)	25 J	26 J	30 J	35 J	25 J	25 J	41 J success
ICD discharge after implant	Present in 2 cases and successful	NR	None	NR	Present and success/fail	None	None	Present and successful	NR	Present and successful	NR	NR	None
Follow-up duration (months)	36 (12–60)	12	14 (3–20)	NR	4	18 (14–24)	6	6	NR	3	NR	NR	12

*Abbreviations:* BMI, body mass index; DFT, defibrillation threshold; F, female; ICD, implantable cardioverter-defibrillator; ICM, ischemic cardiomyopathy; LAX, left axillary vein; LF, left femoral vein; LSC, left subclavian vein; LVEF, left ventricular ejection fraction; M, male; NICM, non-ischemic cardiomyopathy; NR, none reported; RSC, right subclavian vein.

*Maximal generator capacity was 30 J for both cases.

**Table 2:** Clinical Characteristics and Outcomes of 5 Cases Performed at Our Institution

Case	Case 1	Case 2	Case 3	Case 4	Case 5
Age (years)	39	56	62	29	39
Gender	M	M	M	M	M
BMI	31	29	29	54	41
Mean LVEF%	15	35	25	35	33
Etiology of heart failure	NICM	NICM (HCM)	NICM	NICM	ICM
Amiodarone therapy	N	Y	Y	N	N
Left ventricular end-diastolic diameter (cm)	7.7	6.4	6.8	6.7	7.7
Percutaneous access	LAX	LAX	LSC	LAX	LAX
Procedure-related complications	None	None	None	None	None
Maximal DFT tested with prior ICD system	45 J	41 J	41 J	30 J, fail	40 J, fail
DFT after azygos lead insertion (J) with successful cardioversion	35 J	31 J	21 J	28 J, successful	41 J, successful
ICD discharge after implant	None	Present and successful	Present and successful	None	None
Follow-up duration (months)	48	36	12	60	22

*Abbreviations:* BMI, body mass index; DFT, defibrillation threshold; HCM, hypertrophic cardiomyopathy; ICD, implantable cardioverter-defibrillator; ICM, ischemic cardiomyopathy; LAX, left axillary vein; LSC, left subclavian vein; LVEF, left ventricular ejection fraction; M, male; NICM, non-ischemic cardiomyopathy.
